# Gnaq and Gna11 in the Endothelin Signaling Pathway and Melanoma

**DOI:** 10.3389/fgene.2016.00059

**Published:** 2016-04-20

**Authors:** Oscar Urtatiz, Catherine D. Van Raamsdonk

**Affiliations:** Department of Medical Genetics, University of British ColumbiaVancouver, BC, Canada

**Keywords:** GNAQ, GNA11, EDNRB, endothelin, melanoma, melanocyte, GPCR, Schwann cell precursors

## Abstract

In this article, we first briefly outline the function of G protein coupled receptors in cancer, and then specifically examine the roles of the seven transmembrane G protein coupled Endothelin B receptor (Ednrb) and the G proteins, GNAQ and GNA11, in both melanocyte development and melanoma. Ednrb plays an essential role in melanocyte development. GNAQ and GNA11 are oncogenes when mutated in certain types of melanocytic lesions, being extremely frequent in uveal melanoma, which forms from melanocytes located in the eye. Previously, we reported that in mice, Schwann cell precursor derived melanocytes colonize the dermis and hair follicles, while the inter-follicular epidermis is populated by other melanocytes. A pattern has emerged whereby melanocytes whose activities are affected by gain-of-function mutations of the Endothelin 3 ligand and Gα_q/11_ are the same subset that arise from Schwann cell precursors. Furthermore, the forced expression of the constitutively active human GNAQ^Q209L^ oncogene in mouse melanocytes only causes hyper-proliferation in the subset that arise from Schwann cell precursors. This has led us to hypothesize that in Schwann cell precursor derived melanocytes, Ednrb signals through Gα_q/11_. Ednrb is promiscuous and may signal through other G protein alpha subunits in melanomas located in the inter-follicular epidermis.

## Introduction

G-protein coupled receptors (GPCRs) are one of the largest and most diverse membrane protein families, consisting of over 800 members and comprising 30% of drug discovery targets. GPCRs function by detecting a wide spectrum of extracellular signals and ligands, which generate conformational changes in the GPCR structure and cause the activation of signaling networks inside the cell. The non-sensory GPCRs are classified into four main families. These include the rhodopsin-like receptors, which are the most numerous, the secretin-like receptors, the metabotropic glutamate and pheromone receptors, and the frizzled receptors (reviewed in Venkatakrishnan et al., 2013).

The structure of seven transmembrane GPCRs can be divided into three regions: extracellular, transmembrane, and intracellular. The extracellular region consists of the N terminus and three extracellular loops (ECL1–ECL3). The transmembrane (TM) region consists of a core structure of seven alpha helices (TM1–TM7). The intracellular region contains three intracellular loops (ICL1–ICL3), an intracellular amphipathic helix (H8), and the C terminus. The activation of GPCRs begins with the binding of a ligand to the extracellular portions of the receptor, which causes a small conformational change in the TM core. This ultimately leads to larger structural rearrangements at the transmembrane- intracellular domain interface, which alters the interactions of signaling effectors, such as G proteins, GPCR kinases, and arrestins, with the cytoplasmic portions of the receptor. Different ligands produce different conformational states within a GPCR, such as full agonists, partial agonists, inverse agonists, and allosteric modulators. Each of these can bring about different downstream effects. The active state of a GPCR is defined as the conformation of the receptor that couples to and stabilizes an effector molecule [reviewed in (Cabrera-Vera et al., 2003; Venkatakrishnan et al., 2013)].

GPCRs mainly signal through heterotrimeric G proteins, which contain three separate subunits: α, β, and γ. In its inactive state, the α subunit is bound to a guanine diphosphate molecule (GDP) and the β and γ subunits in a complex tethered to the cytosolic side of the cell membrane. When a GPCR is activated by ligand binding, it recruits a heterotrimeric G protein, which triggers the release of GDP and the binding of a guanine triphosphate molecule (GTP) on the Gα subunit. Key residues for GPCR-G protein couplings have been identified within the N termini (McFadzean et al., 1989; Blahos et al., 1998), α2-helices, α2-β4 loop regions (Lee et al., 1995; Onrust et al., 1997), α4-helices, and α4-β6 loop regions (Bae et al., 1997) of the receptor. The GTP-bound Gα subunit dissociates from the receptor and Gβγ subunits to activate downstream effectors. There are five classes of Gα protein (Gα_s_, Gα_q_, Gα_i_, Gα_12/13_, and the newly discovered Gα_v_) and the GTP-bound conformations of each class interact with different canonical downstream effectors. Some GPCRs signal through more than one type of Gα protein, which further increases complexity. In addition, the Gβγ subunits can also act on effectors. The activity of Gα is self-limited by the intrinsic GTPase activity of its Ras-like domain, which hydrolyses GTP back to GDP and prevents further interaction of Gα with its effector. This inactivation step is modulated by Regulators of G-protein signaling (Rgs) proteins, which are GTPase accelerating proteins (GAPs). Some effectors also act as GAPs for Gα, such as the phospholipase C effector for Gα_q_ class α subunits [reviewed in (Conklin and Bourne, 1993; Wess, 1997; Yang et al., 1999; Cabrera-Vera et al., 2003; Oka et al., 2009; Kimple et al., 2011; Sánchez-Fernández et al., 2014)].

## G Protein Coupled Pathways in Cancer

G-protein coupled receptors regulate many key biological functions, such as differentiation, cell proliferation, cell migration, and metabolic activity, thus it is not surprising that GPCRs play a role in tumorigenesis. In general, there are four mechanisms by which G protein coupled pathways drive tumorigenesis: excess ligand availability, excess GPCR expression, activating mutations in GPCRs, and activating mutations in Gα proteins.

Many potent mitogens such as thrombin, lysophosphatidic acid (LPA), gastrin-releasing peptide (GRP), endothelins, and prostaglandins, stimulate cell proliferation by acting on their cognate GPCR in various cell types (Gutkind, 1998; Marinissen and Gutkind, 2001; Rozengurt et al., 2002; Mills and Moolenaar, 2003). In 1991, it was reported that over-expression of muscarinic cholinergic receptors (mAChRs) in NIH3T3 cells was not sufficient for oncogenic transformation. However, when the cells were treated with an excess of carbachol ligand, foci were readily induced. This result showed that normal GPCRs can promote ligand-dependent neoplastic transformation when stimulated by the unrestricted availability of their ligand (Gutkind et al., 1991). In addition, malignancies such as colon carcinoma (Gao et al., 2006), squamous cell carcinoma (SCC) of the lung (Gugger et al., 2008), basal cell carcinoma (Tanese et al., 2008), hepatocellular carcinoma (Yamamoto et al., 2003), prostate cancer (Weigle et al., 2004), breast cancer (Mihai et al., 2006), and glioblastoma multiforme (Shashidhar et al., 2005) have been reported to over-express GPCRs.

Activating mutations in GPCRs represent another tumorigenic route. Mutations in the thyroid-stimulating hormone receptor are found in ∼30% of thyroid adenomas (Parma et al., 1993). Mutations have also been reported in Smoothened (SMO), glutamate metabotropic receptors (GRMs), members of the adhesion family of GPCRs, and receptors for bioactive lipid mediators such as LPA and sphingosine-1-phosphate (S1P) which tend to accumulate in the tumor microenvironment. In 2010, a ground-breaking study revealed an unexpectedly high frequency of somatic mutations in genes encoding GPCRs in breast, lung, ovarian, and prostate cancer, including *LPHN3, GRM8, CMKLR1, MAS1L, AGTRL1*, and *PTGFR* (Kan et al., 2010). It was estimated that 20% of all cancers bear somatic mutations in GPCRs.

Finally, pathway activation can occur through mutations in Gα subunits. Because the Gα subunit must inactivate itself through the hydrolysis of GTP to GDP, mutations that reduce the function of the Ras-like GTPase domain paradoxically lead to constitutive active signaling. In certain cellular contexts, this generates oncogenic transformation. This was first discovered in 1989 when somatic activating mutations in Gα_s_ were found in growth hormone-secreting human pituitary tumors (Landis et al., 1989). Recently, Gα_s_ was reported to be mutated in 3.8% of 28,961 tumor samples, according to the Catalogue of Somatic Mutations in Cancer (COSMIC) database (Forbes et al., 2010). Somatic mutations causing constitutive activity of Gα proteins have been found in other types of cancers as well (Lyons et al., 1990; Van Raamsdonk et al., 2009b, 2010). Common to all these lesions is that the mutations occur in the Ras-like GTPase domain of Gα at the perfectly conserved glutamine and arginine residues that directly contact the gamma phosphate of GTP, stabilizing it for hydrolysis (Landis et al., 1989; Markby et al., 1993; Farfel et al., 1999).

## Gα_q/11_ Subunits in Melanocyte Development and Melanoma

Melanomas arise from pigment producing cells called melanocytes in mammals. Immature melanocytes, melanoblasts, originate in the neural crest, either directly in an early stream, or indirectly from Schwann cell precursors lining developing nerves (Adameyko et al., 2009). During embryogenesis, melanoblasts migrate through the dermis along the dorsal-ventral axis. Some, but not all of the melanoblasts, will enter the epidermis. There they can choose to migrate into hair follicles or persist in between hair follicles in the “inter-follicular” epidermis. Other melanoblasts never enter the epidermis. These non-epithelial melanocytes remain in the dermis or migrate into the uveal tract of the eye or the leptomeninges of the central nervous system.

The majority of human melanomas arise from melanocytes located in the inter-follicular epidermis. The rest arise from non-epithelial melanocytes, which as a group are characterized by frequent oncogenic mutations in the heterotrimeric G protein alpha subunits, *GNAQ* and *GNA11*, which encode Gα_q_ and Gα_11_ (Van Raamsdonk et al., 2009b, 2010; Küsters-Vandevelde et al., 2010) The importance of these two proteins in melanocytes first became apparent during a study of a set of mouse mutants with a darker dermis (*Dsk1*, *Dsk7*, *Dsk10*), obtained during an ENU (N-ethyl-N-nitrosourea) mutagenesis screen of 30,000 mice (Hrabé de Angelis et al., 2000; Fitch et al., 2003; Van Raamsdonk et al., 2004). These mice carried hyper-active, but not constitutively active, single amino acid substitution mutations in either *Gnaq* or *Gna11* at isoleucine 63, valine 179, or phenylalanine 335. These mutations increased the number of melanoblasts in the embryo beginning immediately after the first commitment of these cells to the melanocyte lineage. The increased melanocytes persisted in the dermis throughout the life of the mice, but did not cause tumors. The *Gnaq* and *Gna11* dark dermis mutations acted additively, darkening the dermis in a quantitative and step-wise fashion as the number of mutant *Gnaq* and *Gna11* alleles increased, indicating that the read-out of Gα_q/11_ signaling can be quantitative. Strikingly, the inter-follicular epidermis of the tail, which is pigmented, was unaffected by the mutations, even when all four *Gnaq* and *Gna11* alleles were replaced with gain-of-function *Dsk* versions. The gain-of-function alleles partially rescued the reduction in melanoblast numbers caused by heterozygous loss of the *c*-*Kit* tyrosine kinase receptor, *Pax3* transcription factor, and endothelin B receptor, *Ednrb*. However, homozygous loss of *Ednrb* prevented all Gα_q_ and Gα_11_ generated skin darkening. Based upon previous *in vitro* work (Okamoto et al., 1997; Doi et al., 1999; Imamura et al., 2000), this led to the hypothesis that Endothelin receptor B, a G protein coupled receptor, signals through Gα_q/11_ in melanocytes.

**FIGURE 1:**
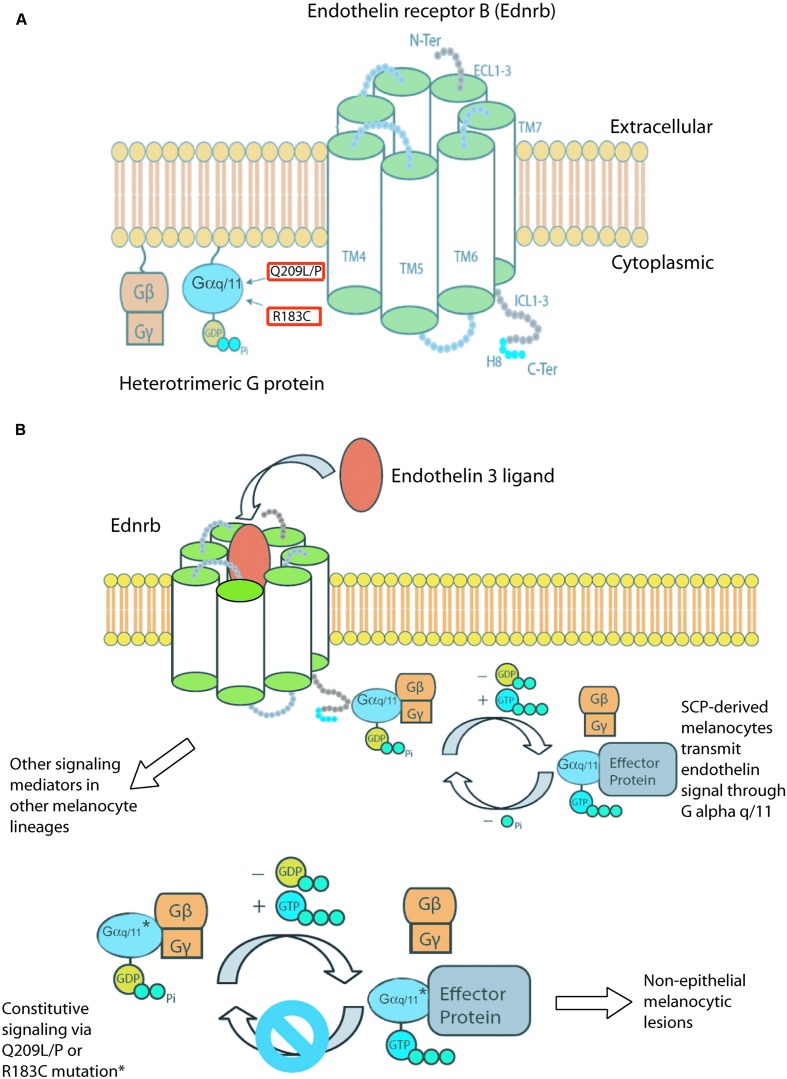
**(A)** Components of the hypothesized Endothelin signaling pathway. Constitutively active mutations found in *GNAQ* and *GNA11* in human melanocytic lesions are boxed in red. **(B)** We hypothesize that Schwann cell precursor derived melanocytes transmit endothelin signals through Gα_q/11_. Schwann cell precursor derived melanocytes are also susceptible to developing melanocytic lesions when either *GNAQ* or *GNA11* are mutated to their constitutively active forms. This melanomagenic pathway gives rise to non-epithelial associated lesions. Other G protein alpha subunits may transmit endothelin signals in epithelial associated melanomas.

The mutations in *GNAQ* and *GNA11* in human melanocytic lesions are somatic, mutually exclusive, and occur at two hotspots, glutamine 209 and arginine 183, which causes constitutive activity. 50%–85% of non-epithelial melanocytic lesions are affected by these mutations, and include lesions in the dermis, called blue nevi, leptomeningeal melanocytomas, uveal nevi, and uveal melanomas. The two genes are mutated with unequal frequency in each type of lesion. *GNAQ* mutations are 7.9 times more frequent in dermal lesions, 4.6 times more frequent in leptomeningeal melanocytomas, and 1.4 times more frequent in primary uveal melanomas, compared with *GNA11* mutations (Küsters-Vandevelde et al., 2010; Van Raamsdonk et al., 2010; Gessi et al., 2012; Küsters-Vandevelde et al., 2014). Conversely, *GNA11* mutations are 2.6 times more frequent than *GNAQ* mutations in uveal melanoma metastases (Van Raamsdonk et al., 2010). Given their presence in uveal nevi, *GNAQ* and *GNA11* mutations are hypothesized to be early events in uveal melanomagenesis (Van Raamsdonk et al., 2010). Q209 mutations are much more frequent than R183 mutations, and are predicted to have a greater inhibitory effect on the GTPase activity of Gα_q_/_11_ (Berman et al., 1996; Orth et al., 2009; Van Raamsdonk et al., 2010) (**Figure 1A**).

Mutations in either gene are extremely rare in human melanomas located in the epidermis. The COSMIC database v72 reported four patients with *GNAQ* mutations among 1,696 entries (0.2%) for superficial spreading, lentigo maligna, nodular, and otherwise unspecified malignant melanomas of the skin. Consistent with this, the forced expression of GNAQ^Q209L^ in mouse melanocytes caused cell loss in the inter-follicular epidermis, in contrast to the hyper-proliferation observed in the dermis, hair follicles, leptomeninges, and uveal tract (Huang et al., 2015). Fate mapping during mouse development has shown that Schwann cell precursor derived melanoblasts migrate to the dermis and hair follicles, but not the inter-follicular epidermis of the tail (Deo et al., 2013). Therefore, we hypothesize that the developmental lineage of a melanocyte determines whether constitutive activity of Gα_q_ and Gα_11_ is oncogenic (**Figure 1B**).

The first effector of Gα_q_ class α subunits to be identified was phospholipase C (PLC). PLC cleaves phospholipid phosphatidylinositol 4,5-bisphosphate (PIP2) into diacyl glycerol (DAG) and inositol 1,4,5-trisphosphate (IP3). IP3 diffuses in the cytoplasm and binds to receptors such as calcium channels in the endoplasmic reticulum, releasing calcium as a second messenger. Calcium and DAG also activate protein kinase C, which can phosphorylate RAF in the MAP kinase pathway. One of the primary effects of oncogenic Gα_q_ and Gα_11_ is the activation of the MAP kinase pathway and the phosphorylation of MEK and ERK (Van Raamsdonk et al., 2009b, 2010). In a randomized, phase II trial of the MEK inhibitor, selumetinib, treatment improved progression-free survival, and the response rate to chemotherapy, but unfortunately did not lead to improvement in overall survival (Carvajal et al., 2014). PKC activity may have additional effects, because PKC inhibition decreased NFkB signaling (Wu et al., 2012) and combined PKC and MEK inhibition is more efficacious at inhibiting MAP-kinase pathway activation, halting proliferation, and inducing apoptosis *in vitro* (Chen et al., 2014). A phase Ib/II combination trial of a MEK inhibitor, MEK162, plus the PKC inhibitor, AEB071, has been initiated (NCT01801358).

MEK inhibition has been reported to lead to PI3K/AKT up-regulation, which may contribute to tumor resistance (Babchia et al., 2010). A phase II trial will compare the MEK inhibitor, trametinib, alone or trametinib plus the AKT inhibitor, GSK2141795 (NCT01979523). Additionally, a phase 1b trial will be launched using the PKC inhibitor sotrastaurin plus the PI3K-alpha inhibitor, BYL719 (Shoushtari and Carvajal, 2014). Alternative effectors mediate Gα_q_ activity independently of PLC, revealing unanticipated complexity. Oncogenic Gαq stimulates the transcriptional co-activator, YAP, through a Trio-Rho/Rac signaling circuitry that promotes actin polymerization, in a pathway that is independent of both PLC and the canonical Hippo pathway (Feng et al., 2014; Yu et al., 2014). This provides yet another rational therapeutic avenue for uveal melanoma. YAP dephosphorylation and nuclear translocation may stimulate melanoma by increasing Notch signaling, through up-regulation of the Notch ligand, JAG-1 (Liu et al., 2015).

## Endothelin Signaling in Melanocyte Development

Endothelin receptors are GPCRs that belong to the rhodopsin-like class. In mammals, there are two Endothelin receptors, type A and B, and three 21 amino acid long Endothelin ligands, Edn1, Edn2, and Edn3, which are processed to their mature forms by Endothelin converting enzymes 1 and 2 (Ece-1, Ece-2). The loss of Endothelin receptor B (Ednrb) in mice causes a severe reduction in melanoblast numbers, while Endothelin receptor A (Ednra) knockout does not produce a pigmentation phenotype (Baynash et al., 1994; Hosoda et al., 1994; Clouthier et al., 1998). Thus, the effect of endothelin signaling in melanocyte development appears to be transduced by Ednrb. Ednrb has an equal affinity for all three of the Endothelin ligands (Sakurai et al., 1990). *Edn3* and *Ece-1* mutant mice have a very similar phenotype compared to *Ednrb* mutant mice, neatly linking the three proteins during development (Baynash et al., 1994; Yanagisawa et al., 1998).

During mouse embryogenesis, Ednrb is expressed in melanoblasts by the time they are committed to the melanocyte cell fate (Lee et al., 2003). In *Ednrb* null embryos, melanoblasts appear in the mesenchyme directly adjacent to the dorsal neural tube, sometimes referred to as the melanoblast staging area, but then are lost in an anterior to posterior progression (Hosoda et al., 1994; Lee et al., 2003). A similar result was found studying Schwann cell precursor derived melanoblasts around cranial nerves IX–X in *Ednrb* null mouse embryos. Melanoblasts appeared in the mutants, but were greatly reduced in number and did not break contact with the nerve (Adameyko et al., 2012). A temporally regulated Ednrb expression system in mice demonstrated that melanoblasts that have migrated into the epidermis do not require Ednrb signaling to persist there (Shin et al., 1999). Thus, the primary role of Ednrb during mouse development seems to be to support and direct newly created melanoblasts when they are migrating in the dermis, which all melanoblasts must do. It is important to note that a few melanoblasts can survive without endothelin signaling in the head and lower body, leading to small patches of pigmented fur in those areas on an otherwise white background (Hosoda et al., 1994). Interestingly, melanoblasts in the midbrain region, which arise independently of nerves, are affected less by *Ednrb* loss and therefore may be a source of pigmented head spots in *Ednrb* mutants (Adameyko et al., 2012). The lack of any pigmentation in the trunk indicates that both Schwann cell precursor derived and other lineages of melanoblasts in that region require Endothelin for development.

In chickens, the function of the endothelin receptor is shared by two different homologs. *Ednrb* is expressed by neural crest precursors prior to commitment to the melanocytic lineage and *Ednrb2* is expressed in migrating melanoblasts (Nataf et al., 1996; Lecoin et al., 1998). Thus, the role of avian *Ednrb2* seems more homologous to mammalian *Ednrb* (Pla et al., 2005). In chick experiments, *Ednrb2* was required for melanoblasts to enter the dorsal-lateral pathway and the ectopic expression of *Ednrb2* in neuronal neural crest cell precursors caused them to switch from the ventral pathway to the dorsal-lateral pathway (Harris et al., 2008). An inverted duplication present in the chicken genome in several breeds leads to the over-expression of *Edn3* (Dorshorst et al., 2012). In these chick embryos, melanoblasts migrate aberrantly along the ventral pathway, in addition to the expected dorsal-lateral pathway (Faraco et al., 2001). This demonstrates that endothelin signaling directs melanoblast migration.

It is not known exactly why melanoblasts fail to persist in *Ednrb* null mouse embryos. Apoptotic melanoblasts were not found in the melanocyte staging area of *Ednrb* mutants, but these cells may have been difficult to detect (Lee et al., 2003) and it is likely that endothelins do stimulate melanocyte survival/proliferation. Many different *in vitro* experiments have shown that Edn3 increases cell proliferation of neural crest cell progenitors and melanoblasts in culture (Reid et al., 1996; Lahav et al., 1998; Dupin et al., 2000; Real et al., 2006). In addition, transgenic over-expression of the Edn3 ligand in the skin increased dermal and epidermal melanoblast numbers during embryogenesis (Garcia et al., 2008). However, melanoblasts in the dermis were more strongly stimulated than melanoblasts in the epidermis. Similarly, the over-expression of *Edn3* in chickens causes intense hyper-pigmentation of the dermis (Dorshorst et al., 2012). In mice, and most other mammals besides humans, melanocytes persist in the post-natal dermis in the tail and ears and, in smaller numbers, in the trunk (Fitch et al., 2003; Van Raamsdonk et al., 2004). These cells probably require on-going endothelin signaling for support (Garcia et al., 2008; Hyter et al., 2013).

## The Relationship Between Ednrb and Gα_q/11_ During Mouse Development

Because homozygous loss of *Ednrb* prevented all Gα_q_ and Gα_11_ generated skin darkening in the *Dsk* mice, it was hypothesized that Ednrb signals through Gα_q/11_ in melanocytes (Van Raamsdonk et al., 2004). Direct data concerning the coupling of Ednrb to a specific Gα subunit in melanocytes is limited. GPCRs can couple to multiple Gα or Gβγ subunits [reviewed in (Hermans, 2003)]. In reconstituted phospholipid vesicles (Doi et al., 1999) and Chinese hamster ovary cells (Okamoto et al., 1997), Ednrb stimulated phospholipase C and inhibited adenylyl cyclase, through Gα_q_ and Gα_i_, respectively. A deletion in the C terminus of Ednrb, and the *Ednrb^G57S^* and *Ednrb^R319W^* mutations, impair Gα_i_ signaling, but not Gα_q_ (Okamoto et al., 1997; Fuchs et al., 2001). In contrast, an *Ednrb^W276C^* mutation impairs Gα_q_ coupling, but not Gα_i_ (Imamura et al., 2000). In human kidney 293 cells, EDN3 increased GTP binding of Gα_13_ (Kitamura et al., 1999). In the cellular context of melanocytes, the treatment of a cell line expressing EDNRB with EDN3 increased inositol 1,4,5-triphosphate and intra-cytoplasmic calcium concentrations, which supports a role for Gα_q_ (Kang et al., 1998). These data high-light the promiscuity of Ednrb.

If all of the effects of Ednrb activation in melanocytes were generated through Gα_q/11_, then the complete knockout of *Gnaq* and *Gna11* would be expected to have the same phenotype as *Ednrb* null mice, i.e., severe hypo-pigmentation. A double knockout of *Gnaq* and *Gna11* is lethal in mid-gestation in mice, however, the knockout of a single allele of *Gnaq* is enough to reduce the pigmentation of the adult dermis (Van Raamsdonk et al., 2004). The presence of melanoblasts was noted at E18.5 in a conditional knockout of *Gnaq* and *Gna11* made specifically in the neural crest lineage, but the number and distribution of these melanoblasts was not described (Dettlaff-Swiercz et al., 2005). A similar conditional knockout of Ednrb in the neural crest lineage eliminated melanocytes in the trunk (Druckenbrod et al., 2008).

There are several possible explanations for why *Gnaq* and *Gna11* conditional knockout in the neural crest lineage did not completely eliminate melanoblasts. One is that Ednrb does not couple to Gα_q/11_ in melanoblasts and the similarities in the effects of *Edn3* over-expression and Gα_q/11_ hyper-activity are just coincidental. We think this is unlikely. Another explanation is that in the absence of Gα_q/11_, Ednrb receptors activate other G protein alpha subunits, which buffers the system. A third possibility is that with four alleles of *Gnaq* and *Gna11*, Cre is not as effective at eliminating all Gα_q/11_ as compared to Ednrb. There is no data yet to distinguish between these possibilities.

However, in yet another striking similarity, both the over-expression of *Edn3* and the hyper-active *Dsk* alleles of Gα_q/11_ darken the mouse coat through increased post-natal pigment production in hair follicles, using a mechanism that is independent of melanocyte cell number (Garcia et al., 2008; Van Raamsdonk et al., 2009a). *Edn3* expression also correlates with light and dark areas of cat coat color (Kaelin et al., 2012). We point out that Schwann cell precursor derived melanocytes localize to the dermis and hair follicles, and these are both places where Edn3 and Gα_q/11_ gain-of-function mutations generate developmental phenotypes. Meanwhile, the inter-follicular epidermis is virtually unchanged as a result of hyper-active Gα_q/11_ (Van Raamsdonk et al., 2004) and Schwann cell precursor derived melanocytes do not colonize this part of the epidermis (Deo et al., 2013). Thus, there is a correlation between melanocyte lineage, response to Edn3 and Gα_q/11_ gain-of-function mutations, and melanocyte location during development (**Figure 1B**).

## The Relationship Between Ednrb and Gα_q/11_ in Melanomagenesis

Gα_q/11_ mutations play an obvious role in the development of non-epithelial associated melanocytic lesions. There are specific hotspot oncogenic mutations that are very frequently found in a specific subset of melanocytic lesions, which do not involve the epidermis. The role of Ednrb in melanoma is more difficult to interpret. In fact, much of the data concerns the effects of Ednrb on melanomas located in the epithelium, which implies that the effects of Ednrb are not a result of Gα_q/11_ activation. As Ednrb is probably able to couple to multiple G alpha subunits, this is perhaps not surprising. In addition, signaling pathways do not play the exact same roles in development as they do in cancer, even while cancer often reactivates innate developmental pathways. We will first summarize what has been reported about Ednrb signaling in melanomas associated with the epithelium, then discuss findings in uveal melanoma.

In general, EDNRB expression is positively correlated with melanoma progression in cutaneous melanoma (Demunter et al., 2001). Over-expression of EDNRB increased melanoma brain metastases in mice orthotopically transplanted with human melanoma cell lines (Cruz-Muñoz et al., 2012). The activation of EDNRB by endothelins induces the expression of HIF-1α, which leads to the up-regulation of vascular endothelial growth factor and its receptor in primary and metastatic melanoma cell lines, resulting in MAP kinase and AKT activation (Spinella et al., 2012). These pathways up-regulate MCAM, a melanoma cell adhesion molecule that promotes invasion and metastasis (Williams et al., 2014).

The EDNRB inhibitors, BQ788, A192621, and Bonsentan inhibit melanoma cell growth *in vitro*, in xenografts of human melanomas in nude mice, and in some human patients, and restore cell morphology to a more normal appearance (Bagnato et al., 2004; Lahav et al., 2004; Berger et al., 2006; Kefford et al., 2007; Cruz-Muñoz et al., 2012; Asundi et al., 2014; Wouters et al., 2015). One recent study found that using a combination of an antibody-drug conjugate targeting EDNRB, together with small-molecule inhibitors of the MAP kinase pathway, increased anti-tumor activity in BRAF/NRAS mutant cell lines and tumor models (Asundi et al., 2014).

In contrast to these studies, putative loss-of-function germline variants in *EDNRB* were associated with cyclin-dependent kinase inhibitor 2A, *CDKN2A*, variants in familial melanoma cases in a French population, but not in an Italian population (Spica et al., 2011). *EDNRB* variants were not associated with sporadic melanoma in either the French or Italian populations. Larger studies and other populations are necessary to validate these findings. Endothelin signaling has also been suggested to enhance nucleotide excision repair following UV damage, and so a deficiency in this process could possibly act synergistically with a weak checkpoint (D’Orazio, 2015; von Koschembahr et al., 2015).

Next, we will consider uveal melanoma. Although *EDNRB* mutations have not been identified in uveal melanoma, the loss of *EDNRB* expression in uveal melanoma has been correlated with a worse prognosis in two different studies (Smith et al., 2002; Onken et al., 2004). Because oncogenic Gα_q_/_11_ mutations increase signaling, this is seems unexpected and contradictory if one assumes that EDNRB signals through Gα_q_/_11_ in uveal melanoma cells. However, there are two important points to consider. One, it is not known yet whether Gα_q_/_11_ proteins with constitutively active mutations require a receptor for activation or, in fact, will even couple to a receptor given that they are fixedly bound to GTP. EDNRB and Gα_q_/_11_ may become unlinked in the situation of uveal melanoma. If this is true, then it is possible that a decrease in the expression of EDNRB may reduce activation of the wildtype Gα_q_/_11_ proteins, which could somehow enhance the effect of the mutant Gα_q_/_11_ proteins through a lack of competition for downstream effectors.

The second point is that uveal melanoma patients without *GNAQ* or *GNA11* mutations tend to have a worse prognosis than those with *GNAQ* or *GNA11* mutations (Van Raamsdonk et al., 2010). In a completely different hypothesis, *GNAQ* and *GNA11* mutations could be early events that boost melanocyte cell growth, but eventually it is necessary to down-regulate Gα_q_/_11_ signaling to achieve metastasis. This could be accomplished by down-regulating *EDNRB* expression. Melanocytes without *GNAQ* and *GNA11* mutations would not need to go through this step, having arisen through a different mechanism, and thus progress more rapidly. In the future, more information on the interactions of EDNRB with different mutant and wildtype G protein alpha subunits is needed. In addition, it would be very interesting to correlate tumor progression with both *EDNRB* expression and the presence or absence of *GNAQ* and *GNA11* mutations in uveal melanoma.

## Conclusion

During development, both Ednrb and Gα_q/11_ regulate melanoblasts migrating in the dermis with strikingly similar effects. This suggests that they are closely linked together in the same signaling pathway. In support of this, Ednrb is required for the hyper-proliferative effects of the hyper-active *Dsk Gnaq* and *Gna11* alleles. Ednrb is also required for the development of almost all melanocytes in the trunk, because all melanoblasts must migrate through the dermis, even if their eventual destination is the epidermis. Gain-of-function mutations in Edn3 and Gα_q/11_ increase melanocytes in the dermis and have little effect on those in the inter-follicular epidermis. In addition, gain-of-function mutations in Edn3 and Gα_q/11_ darken coat color using a mechanism that is independent of cell number in hair follicles. Thus, a pattern has emerged whereby melanocytes whose activities are affected by gain-of-function mutations of the Endothelin 3 ligand and Gα_q/11_ are the same subset that arise from Schwann cell precursors. Furthermore, the forced expression of the human GNAQ^Q209L^ oncogene in mouse melanocytes only caused hyper-proliferation in the subset that arise from Schwann cell precursors. This has led us to hypothesize that in Schwann cell precursor derived melanocytes, Ednrb signals through Gα_q/11_. Ednrb is promiscuous and may signal through other G protein alpha subunits in melanomas located in the inter-follicular epidermis.

## Author Contributions

All authors listed, have made substantial, direct and intellectual contribution to the work, and approved it for publication.

## Conflict of Interest Statement

The authors declare that the research was conducted in the absence of any commercial or financial relationships that could be construed as a potential conflict of interest.
